# UTILIZING NUTRITION SERVICES PROVIDERS TO PREVENT LATE-LIFE SUICIDE: IMPLEMENTING A RANDOMIZED, CONTROLLED TRIAL

**DOI:** 10.1093/geroni/igz038.3039

**Published:** 2019-11-08

**Authors:** Matthew C Fullen, Mary Chase Mize, Laura R Shannonhouse

**Affiliations:** 1 Virginia Tech, Blacksburg, Virginia, United States; 2 Georgia State University, Atlanta, Georgia, United States

## Abstract

A challenge in preventing late-life suicide is identifying and responding to persons-at-risk prior to a suicide attempt. Distressed older adults are less likely to turn to a mental health professional, meaning that community-based prevention strategies are vitally important to comprehensive prevention frameworks. Due to their “natural helper” role, nutrition services (NS) volunteers may be well-positioned to identify suicide warning signs and respond accordingly. Unfortunately, there is a lack of systematic, empirically-tested evaluations of the effectiveness of community-based strategies to prevent older adult suicide, including the use of NS volunteers. To remedy this, the authors partnered with several home- and community-based service organizations to measure the impact of training nutrition services volunteers in suicide prevention skills. The authors will present preliminary findings from this federally-funded randomized, controlled trial of suicide prevention training (i.e., ASIST; safeTALK) on late-life suicidality and its correlates.

